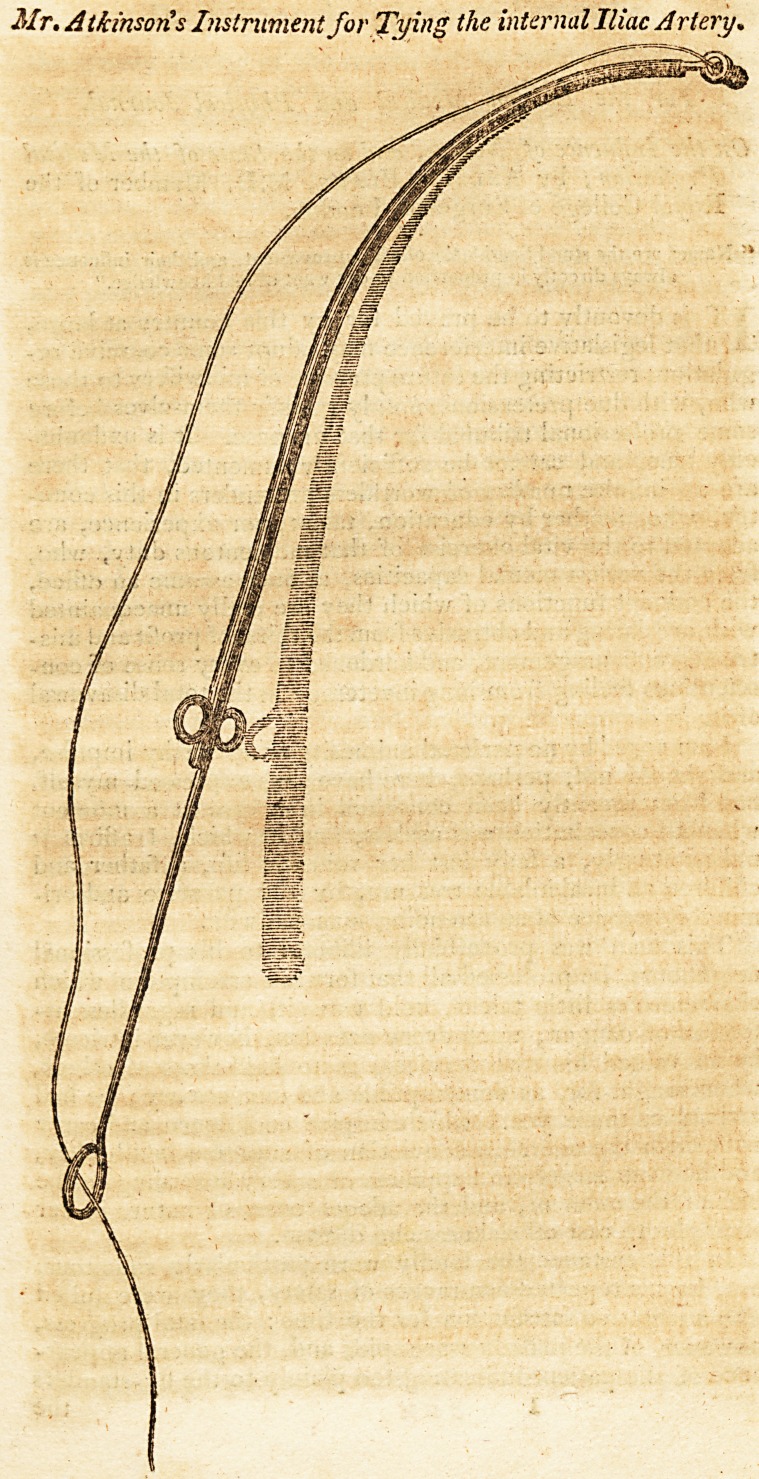# On Tying the Internal Iliac Artery

**Published:** 1817-10

**Authors:** James Atkinson

**Affiliations:** Surgeon to his Royal Highness the Duke of York, Senior Surgeon to the York County Hospital and Dispensary.


*For the London Medical and Physical Journal.*

### *On Tying the internal Iliac Artery;* by James Atkinson, Esq. Surgeon to his Royal Highness the Duke of York, Senior Surgeon to the York County Hospital and Dispensary.

(With an Engraving.)

THE operation of tying the internal iliac artery, the subject of this paper, had been performed for the first time, as far as was known in any country, by Mr. Stevens, of the island of Sta. Cruz, on the 27th of December, 1812, upon a negress-slave from Banbara. It was attended with complete success; and the greatest praise is due to him for the skill, novelty, nerve, and good management of the case.

In absolute deference to the public, I offer the recital of an unsuccessful repetition, under my hands, of this interesting operation. I offer it as a land-mark of practice, to be accepted, amended, or avoided, by those practitioners who in future may be compelled to travel that way. We are all more or less ashamed (and, perhaps, ought to be) of the want of success in those instances where we presume upon our skill, or put our adroitness to the trial. I shall, upon this occasion, cheerfully submit to whatever imputation a wise medical public may lay to my charge. The motive for my undertaking it, was pressing, was vital;—I neither braved it spontaneously, nor did I abandon the operation,—for pain and circumstances were urgent, the tumour was ready to burst,—in surgical truth, the man was dying. So situated, (fatal as was the instance,) the patient's death in reality was suspended; in other words, his life was prolonged by it.

The following is the case:—Thomas Cost, aged 29, presented himself at the York County Hospital, April 29th.

 He

He was a tall, strong, active bargeman, not corpulent, but of marked muscle. He was enduring great pain from a large renitent pulsating tumour situated under the glutæus of the right side,—an obvious aneurism. It had existed about nine months, and was the consequence of a blow from a stone. In hopes of abating tension, and of palliating pain, it was deemed right for a while to enjoin strict quiet, and to use a local application. For three or more days it had the effect, but a recurrence of the symptoms drove me to an immediate consultation. Dr. Lanson, Dr. Wake, and myself, (then present at the Hospital,) saw no alternative but death or the operation.

The novelty, danger, and want of experience, in the practice and performance of it, were duly considered. Loss of reputation on my side, absolute possibility of failure in the execution, and such like feelings, presented themselves to us. But it appeared a duty to wave these considerations, when the chance of preserving a fine young man, in the vigour of life, with a dependant wife and child, were put into the balance. May I, therefore, hope to find justification on this point, for an attempt which had once succeeded, and was, in our opinion, the one thing needful.

The operation was performed on the 12th of May last, without any material difficulty or interruption, except such as was the consequence of the division of, and discharge from, the small muscular arteries, in a subject where they had been much used and exerted, in his occupation of bargeman. Having got command of the internal iliac artery, within the pelvis (which required the complete length of the fingers to accomplish), it was tied. Sufficient proof of its being the identical artery was repeatedly taken,—by the absolute and sensible command it afforded me over the pulsation; and by as repeated evidence of the simultaneous subsidence of it, on pressure. Dr. Wake, Mr. Ward (the house-apothecary), and all the pupils, were requested to be assured beyond a doubt of this circumstance, on repeated trials.—The artery being then tied, Dr. Wake keeping his fingers and hand upon the tumour, we were then again perfectly satisfied of the pulsation being stopped—completely stopped, as far as sense could justify the perception. I could also very distinctly ascertain the interruption of it, by having the artery so decidedly within my fingers, and as much so as in any operation for popliteal or other aneurism which I had ever performed; whereas, in traversing the external iliac, I was sensible of an evident, though feeble, pulsation. Some delay was occasioned by the inaptness of the needle, needle, which was not sufficiently pliable in passing it around the artery. It happened to be one which I had used on other occasions, and a particular instrument I had ready prepared was, by an accident, unfortunately rendered of no avail to me.

The practical analysis of the above case may comprise two periods—the first, or the immediate stage from the operation (a time of truce); the second, when the consequences of it had a threatening aspect, i. e. when the discoloured discharge began to forewarn us of the probable and fatal termination.

During the former period, all was calm and encouraging; the face was occasionally somewhat flushed, but no other sinister symptom was present. The man no longer suffered pain, having been instantly relieved from it, and from the pulsation by the operation; and the tumour was soon very sensibly diminished. His cry was for food, and to be permitted to get up. The pulse, I think, never exceeded 130; and after awhile sunk to about 85 or 90, not exceeding it until the discharge of pus, serum, ichor, or of more fluid blood, exhausted and destroyed him.

In the first instance he continued many days without any pain, or, as he expressed it, as easy as ever he was in his life. The discharge, when it did commence, and apparently only from the ligatures of the skin, (which, in future, I would not make use of,) set in kindly, increasing, however, by degrees in quantity, and deteriorating in quality. After the discoloured discharge had commenced, which was about eight or ten days, I think, before his decease, it was followed by the extrusion of small lumps of conjesta, and of coagulum, which grew more and more abundant, but retaining, for some days, that character. As his dissolution, however, approached, it had a more florid and fluid appearance, and once or twice indicated a more recent and copious hæmorrhage: still he was not visited by faintings or deliquia, so consequent on active bleeding.

He died quietly, and completely of exhaustion, on May 31st, wanting two days of three weeks from the time of the operation.

The medical treatment was simple, and chiefly directed to keeping the bowels in order.

Being uncertain whether he might not be soon removed from the hospital, by his friends, for interment, I lost no time in opening the body. Many circumstances made me impatient so to do, which will be readily anticipated by any person in the habit of performing either interesting or novel operations.

operations. I requested Dr. Wake* to do me the favour of attending the examination.

We first opened the abdomen, to ascertain the state of its parietes, of the peritonæum, and of the general cavity. There was not any appearance of disease—it was whole and uniform, with the exception of an unimportant degree of thickening, and some slight adhesion of the head of the colon and ileum to the adjacent parts. This uniformity was very apparent, not only to Dr. Wake and to myself, but to Mr. Ward (who had paid the greatest attention to the patient) and to the pupils of the hospital present.

I proceeded to re-open the external wound, which had completely healed (saving where the ligature had protruded), and so to examine the course of the operation.

The cavity on the external part of the peritonæum (the seat of the incision) was completely filled with a grumous pillar, or thrumbus of blood, strong and tenacious. The ligature, on moving a part of this with the sponge, readily followed it, and, without doubt, had been disengaged for some days. The knot of the ligature included a small fragment of (apparently) the artery, which projected in a very trifling degree on each side of it. There was a small portion of coagulum under the cartilages of the ribs; and the pillar reached down to the lower seat of the ligature within the pelvis.

We next turned our attention to the artery, as the subject of the operation. For this purpose, the best mode appeared obviously to dissect and clear the aorta within the abdomen, at a certain distance above the bifurcation; to expose the external and internal iliacs, first on the sound side, then on the diseased, which, being accomplished, we had both sides in open view. We then passed a bougie into the external iliac on the sound side, and down the internal on the same side, to ascertain its direction; and then also passed an instrument into the external iliac on the operated or right side, down to the groin, and there left it. We also passed another into the internal iliac, to the part where it had been tied. The internal iliac being exposed, we found that it had been tied, and had separated about an inch from the bifurcation with the external iliac. It was broader, flatter, and more enlarged up to the external iliac, than the external iliac of the same side, or the internal of the opposite. This

* This gentleman, I am happy to express, assisted me both during the operation and the whole course of the business, in the most honourable, anxious, and friendly manner.

circumstance circumstance had been obvious to me whilst I was endeavouring to separate it during the operation, and which I noticed at the time,—for a degree of thickening and of adhesion of its coats was very evident, although it required little force to pass and perforate the internal adhesion of the sides of this artery; and therefore the inside of the mouth or opening, and sides of the internal iliac above where the ligature had been applied, on separating them, had a granulous and greyish coagulated appearance.

The exposure of the respective arteries at the same time on each side, exhibited at once a very clear and satisfactory view of the state of the parts.

A certain space of separation was visible betwixt the upper and lower parts of the internal iliac artery, and now obviously presented to view.

In the lower part of it the artery was wider, and the coats evidently more diseased; and a coagulum of grumous blood was manifest at the mouth of the lower artery, which, being gently removed, exposed this (now more open) mouth, which again, after a short interval, was filled with more fluid ichor from the sac below.

Thus much being satisfactorily ascertained by all present, it was thought expedient to open the glutæal or aneurismal sac, confined to the nates.

It was effected by a free incision, which, dividing the muscle, soon exposed a more membranous surface, the actual sac of the aneurism. From it I cleared away some pounds of coagulum, moistened by a thinner ichorous fluid, and sponged it well, to make all the parts as distinct as their state would permit. I could not perceive any collateral artery opening into it; but a bougie, passed into the lower diseased artery, within the pelvis, in a line with the superior one, was slowly introduced, and easily made way into the exposed sac, from whence the blood had been cleared;—so that it became obvious that a regurgitation of blood might easily happen, and which, no doubt, was the case, from the sac up the artery into the pelvis, at a given time after the ligature had separated.

The resistance from so large a tumour of blood *a tergo*, confined as it was by the sac, and aided by the pressure and impetus of powerful muscular action, must, I apprehend, force the blood by necessity into any aperture, *quâ datâ portâ;* so that a regurgitation of blood upwards would, in my opinion, possibly supplant, in this instance, the usually supposed effect of aneurism, or a bursting of the sac. In each way, however, it is probable that death would equally be the result to the unfortunate patient.

The

The state of the parts having been thus satisfactorily proved, the intent of the enquiry respecting the completion of the operation of tying the internal iliac artery was most unequivocal.

Whether the operation, under the circumstances of the size and tension of the parts, or duration of the disease in so strong a man, should or not have been undertaken, I most implicitly submit to future arbitration. I confess I have many doubts of the propriety of it under such conditions.

Had it been where an incipient tumour was arising, where little pain or pulsation were present, where the subject was (like the negress) very spare and much reduced, the circulation at a low ebb, or where the circulating vessels had not been much dilated by strong labour, I should not make a moment's hesitation, if other means were not adviseable. And, should such a case again occur to me, I shall reflect awhile upon the propriety of opening the external tumour some days after the artery has been tied, provided it should be larger than expedient for operating. The riddance of the coagulum in such a case might possibly avert the fatal effect of the blood seeking its retrograde passage up the diseased artery. This effect strikes me to have been the main cause, in this instance, of the loss of my patient.

I refer and believe the above account to be a correct and circumstantial detail of this case; and must apologize for the length of it.

We have many instances, in extenuation, within the scope of our practice on the casualties of our art, to assure us that there is scarcely an artery in the body which may not be tied with reasonable hope of success, provided it be fairly attainable, and can be commanded by the operator—such are the wonderful resources of our nature!

*York; August* 9, 1817.

P.S.—Perhaps it may be useful to know that the instrument (as I apprehend it would promptly have answered) consisted of a pliant silver wire, with a small ring at the end. The wire passed through a small bent silver tube, or catheter. A ring was added, and to this the ligature was fixed, withoutside the tube. Thus the wire could then have been thrust forth with the ring, when the catheter had been passed round the artery, and, in this way, would have been easily taken hold of by the operator or his assistant; and the wire, being larger than the catheter, would have projected to any due distance for his convenience.

The instrument should be about an inch and a half longer than the following drawing, which is by one of my children, and may be more explanatory and convenient.

*Mr. Atkinson's Instrument for Tying the internal Iliac Artery.*

## Figures and Tables

**Figure f1:**